# Metabolic and clinical markers of T2D in obese Indians: an age-sex matched case-control study

**DOI:** 10.3389/fcdhc.2026.1809646

**Published:** 2026-08-05

**Authors:** Anagha Vyawahare, Pramod Tripathi, Nidhi Kadam, Diptika Tiwari, Baby Sharma, Thejas Kathrikolly, Malhar Ganla, Banshi Saboo

**Affiliations:** 1 Research Department, Freedom from Diabetes Research Foundation, Pune, Maharashtra, India; 2 Department of Management, Freedom from Diabetes Clinic and Research Foundation, Pune, Maharashtra, India; 3 Department of Medicine, Dia Care - Diabetes Hormone Clinic, Ahmedabad, Gujarat, India

**Keywords:** Indian population, matched case-control study, obesity, risk stratification, type 2 diabetes, beta cell function, insulin resistance, temporal internal validation

## Abstract

**Introduction:**

Type 2 diabetes (T2D) is a significant metabolic disorder with disproportionately high burden in obese South Asian populations, yet not all obese individuals develop the disease. This study identified the metabolic, inflammatory, and pharmacological markers associated with prevalent T2D in obese Indians using age- and sex-matched case-control analysis.

**Methods:**

A matched-pair analysis was performed on 514 age- and sex-matched pairs with and without T2D. Patients had obesity [body mass index (BMI) 25–45 kg/m², aged 20–75 years] and participated in a one-year lifestyle intervention program in India. Anthropometric, biochemical markers and medical history were analyzed. Homeostatic model assessment for insulin resistance (HOMA2IR) and beta-cell function (HOMA2%B) was calculated. Significant markers were incorporated into a logistic regression model and temporally validated in an independent time-cohort (n=966). Performance was evaluated through discrimination [receiver operating characteristics area under the curve (ROC-AUC)], calibration, and decision curve analysis. A nomogram was constructed based on the logistic regression model.

**Results:**

Median age and BMI were 46 (IQR 12) years and 29.7 (IQR 5.5) kg/m². Key markers associated with prevalent T2D were poor beta-cell function (HOMA2%B ≤50; OR = 40.9, 95% CI: 24.5–68.2), insulin resistance (HOMA2IR ≥2; OR = 4.5, 95% CI: 3.0–6.7), low HDL-C (OR = 2.1, 95% CI: 1.5–3.0), elevated hsCRP ≥3 mg/L (OR = 1.9, 95% CI: 1.4–2.7), Obesity Class I (BMI 25.0–29.9 kg/m²; OR = 2.1, 95% CI: 1.5–2.9), and antihypertensive medication use (OR = 1.7, 95% CI: 1.2–2.4). Model discrimination was good (AUC 0.860 development, 0.865 validation, both p<0.001). The Hosmer-Lemeshow test showed good model fit (p=0.562). Isotonic-calibrated Models 1 and 2 had Brier scores 0.14 and 0.162, indicating acceptable calibration. Decision curve analysis confirmed net clinical benefit across threshold probabilities 0.10–0.85.

**Conclusion:**

This study identified and temporally validated key metabolic and clinical markers associated with prevalent T2D, demonstrating good discrimination (AUC 0.860) in obese Indian individuals. External validation and prospective evaluation are needed before clinical application.

## Introduction

Type 2 diabetes mellitus (T2D) is a chronic metabolic condition characterized by persistent hyperglycemia resulting from progressive insulin resistance, beta-cell dysfunction, or both (1). With over 500 million individuals affected globally, T2D poses a disproportionate burden in South Asian populations, including Indians, who face heightened susceptibility due to genetic predisposition, sedentary lifestyles, and rapid nutritional transition (2). The rising global prevalence of obesity projected to affect more than half the world’s population within the next decade (3), is a principal driver of this epidemic, as excess adiposity promotes both insulin resistance and beta-cell impairment, substantially increasing diabetes risk (4, 5). Chronic low-grade inflammation has also been implicated in the transition from obesity to T2D, alongside cardiometabolic comorbidities such as hypertension and dyslipidemia (6, 7).

Despite the well-established link between obesity and T2D, not all obese individuals develop the condition. A subset, termed ‘metabolically healthy obese’, maintains near-normal glucose regulation despite comparable adiposity and BMI (8). While insulin resistance has traditionally been regarded as the primary metabolic driver of T2D in obese individuals, accumulating evidence highlights beta-cell dysfunction as an equally important determinant (5). This is particularly relevant in South Asian populations, who demonstrate greater visceral adiposity and impaired beta-cell reserve at lower BMI thresholds compared to other ethnic groups (9). Indians, in particular, exhibit higher insulin resistance and poorer beta-cell function from an earlier age, placing them at substantially elevated risk of T2D even at modest degrees of obesity (2, 9).

Given these population-specific metabolic characteristics, robust risk stratification tools tailored to obese Indian individuals are needed. Most existing models for T2D risk assessment have been developed in the general population, without specific focus on individuals already at elevated metabolic risk due to obesity (10, 11). While both insulin resistance and beta-cell dysfunction have been identified as important determinants of T2D in general population studies (12, 13), their relative contributions in obese Indian individuals remain inadequately characterized. This knowledge gap limits the development of precise discrimination tools and targeted preventive interventions for this high-risk group.

The present study therefore aimed to identify and temporally validate metabolic, inflammatory, and pharmacological markers associated with prevalent T2D in obese Indians using an age- and sex-matched case-control design. Temporal internal validation was performed using an independent time-cohort from the same institution (September 2023–January 2025) to assess the discriminative stability of identified markers. A clinical nomogram was developed to support individualized risk stratification in clinical practice. These findings aim to address a critical gap in the Indian diabetes literature and inform targeted prevention strategies for obese individuals at high risk of T2D.

## Materials and methods

### Study design and population

This retrospective cross-sectional study examined data from patients with obesity (BMI 25–45 kg/m², aged 20–75 years) enrolled in a structured one-year online lifestyle intervention program at Freedom from Diabetes, Pune, India, between June 2020 and August 2023. A total of 514 age- and sex-matched case-control pairs (n=1, 028) were identified, comprising individuals with and without T2D.

T2D diagnosis was based on at least one of the following criteria, consistent with American Diabetes Association (ADA) 2023 guidelines (14): (1) fasting plasma glucose ≥126 mg/dL (7.0 mmol/L) on two separate occasions; (2) HbA1c ≥6.5% (48 mmol/mol) confirmed on repeat testing; or (3) a documented diagnosis of T2D in clinical records by a qualified physician, including patients currently on antidiabetic pharmacotherapy.

### Eligibility criteria

**Figure 1:**
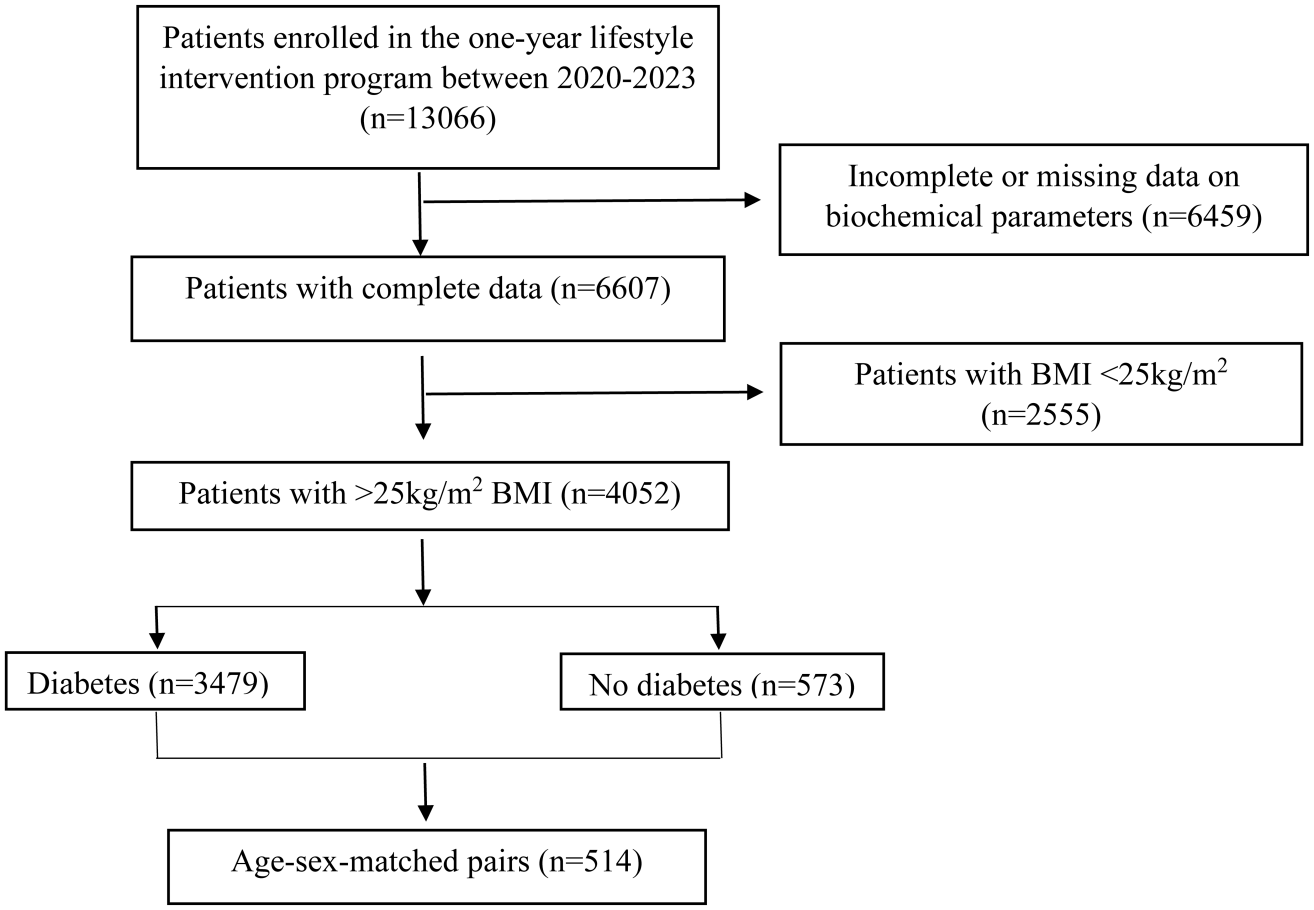
Flowchart of the selection procedure of patients for the study.

Cases were individuals meeting the T2D diagnostic criteria with BMI 25–45 kg/m² and complete first-consultation data. Controls were individuals with no diabetes diagnosis, matched 1:1 to cases by age (± 5 years) and sex. Individuals were excluded if they had BMI <25 kg/m², a diagnosis other than T2D (type 1 diabetes mellitus, maturity-onset diabetes of the young, latent autoimmune diabetes in adults, or gestational diabetes), were on external insulin therapy, or had incomplete baseline data. The patient selection flowchart is presented in **Figure 1**.

Of 13, 066 patients screened, 6, 459 (49.4%) were excluded due to missing values at first consultation, predominantly fasting insulin and hsCRP, which are specialized investigations not universally completed at initial presentation, unlike standard parameters (HbA1c, fasting glucose, lipid profile, BMI) which are collected routinely at enrolment. A further subset had these values recorded at subsequent consultations; only first-consultation values were used to capture pre-intervention baseline metabolic status, as later values would be influenced by the lifestyle intervention.

### Ethical clearance

This study was approved by the Institutional Ethics Committee (Ref. No. FFDRF/IEC/2024/7) and registered with the Clinical trials registry of India (Ref. No. CTRI/2024/03/064596). The requirement for informed consent was waived by the ethics committee owing to the retrospective nature of the study. This study followed the ethical principles outlined in the Declaration of Helsinki.

### Anthropometric and biochemical measurements

Data extracted at first consultation included age, sex, anthropometric measurements (height, weight), biochemical parameters (HbA1c, fasting blood glucose, fasting insulin, lipid profile), blood pressure (systolic and diastolic), and medical history (diabetes duration, comorbidities, medication use). Hypothyroidism was identified by current levothyroxine prescription at first consultation. BMI was Classified using WHO Asian-specific cut-offs (15): Obesity Class I (BMI 25.0–29.9 kg/m²) and Obesity Class II (BMI ≥30.0 kg/m²). These cut-offs follow WHO Asian-specific recommendations, where BMI ≥25.0 kg/m² is considered obesity a threshold lower than the global WHO cut-off of ≥30.0 kg/m² (15). Insulin resistance and beta-cell function were assessed using the HOMA2 calculator (16). HOMA2IR ≥2 indicated insulin resistance (17) and HOMA2%B ≤50 indicated poor beta-cell function (18). Low HDL-C was defined as <40 mg/dL in males and <50 mg/dL in females (19). Systemic inflammation was defined as hsCRP ≥3 mg/L (20).

### Validation dataset

Temporal internal validation was performed using an independent time-cohort of patients (n=966) with obesity (BMI 25–45 kg/m²) enrolled between September 2023 and January 2025, adhering to the same eligibility criteria but without age-sex matching. No patients from the development cohort were included in the validation dataset.

Matching was not applied to the validation cohort as the purpose of temporal internal validation is to assess the discriminative stability of identified markers in an independent time-cohort, rather than to control confounding as in the development phase. The unmatched design better reflects real-world clinical populations and provides a more stringent test of marker stability. Structural differences between cohorts (age, sex distribution, diabetes duration) are acknowledged and systematically documented in **Supplementary Table 1**.

### Post-hoc power calculation

*Post-hoc* power was calculated using the method of Kelsey et al. (21) for 1:1 matched case-control designs (discordant pair approach), implemented in Python v3.8 (scipy.stats library), with α=0.05 (two-tailed) and n=514 matched pairs. Power exceeded 85% for five of seven predictors at observed prevalences. Hypothyroidism (power=66.5%) and Obesity Class I (power=4.4% bivariate) were lower, the latter attributable to near-identical Obesity Class I prevalence in both groups consistent with the obesity paradox finding discussed later in discussion. The retrospective design determined sample size by data availability; the *post-hoc* calculation confirms adequate power for all five primary significant predictors.

### Statistical analysis

Statistical analyses were performed using IBM SPSS v21, R, and Python v3.8. Categorical variables are presented as frequencies and percentages; continuous variables as median (interquartile range) based on skewed data distribution. The McNemar test assessed associations between categorical variables, and the Wilcoxon signed-rank test compared continuous variables between matched pairs. Significant factors from univariate analysis (inflammation, insulin resistance, beta cell function, HDL-C, antihypertensive medication, hypothyroidism, and BMI) were included in conditional logistic regression to estimate adjusted odds ratios (ORs) accounting for the matched-pair structure. Binary logistic regression was subsequently applied to generate absolute predicted probabilities required for discrimination, calibration, decision curve analysis, and nomogram construction, as conditional logistic regression cannot generate predicted probabilities due to the absence of an intercept term (22).

Given the near-complete separation observed for HOMA2%B where poor beta-cell function was present in 285 of 514 diabetic patients (55.4%) but only 19 of 514 non-diabetic patients (3.7%), Firth penalised logistic regression was additionally performed as a confirmatory sensitivity analysis. Firth’s method applies a Jeffreys invariant prior penalty to the likelihood function, producing bias-corrected coefficient estimates that are stable under separation conditions. As the firthlogist package is incompatible with Python 3.12, Firth regression was implemented using the Jeffreys prior penalty using logistf package, R v4.5.3. Results were compared against standard and conditional logistic regression estimates to confirm consistency of findings.

Variance Inflation Factors (VIF) confirmed no multicollinearity among predictors (all VIF <1.1: HOMA2%B=1.06, HOMA2IR = 1.03, HDL-C=1.02, hsCRP=1.01, antihypertensive=1.03, hypothyroidism=1.02, BMI = 1.01).

Binary logistic regression was repeated on the validation dataset (n=966) using the same markers identified in Model 1 to assess discriminative strength in the independent time-cohort. Results are presented as OR with 95% confidence intervals (CI); statistical significance was set at p<0.05.

### Discrimination, calibration, and decision curve analysis

Model discrimination was assessed using the area under the receiver operating characteristic curve (AUC ROC), interpreted as: poor (0.5–0.7), acceptable (0.7–0.8), good (0.8–0.9), and excellent (≥0.9) (23). Calibration was evaluated using the Hosmer–Lemeshow goodness-of-fit test (p>0.05 indicating good fit) (24), the Brier score (lower values indicating better calibration), and calibration plots (Python v3.8). Isotonic regression was applied as a smoothing technique to reduce bin-level noise in the calibration curve visualization. Clinical utility was assessed using decision curve analysis (DCA), estimating net benefit across threshold probabilities (Python v3.8) (24). Internal validation was performed using bootstrap resampling (1, 000 resamples, SPSS v21). Blood pressure data were available for 761 of 1, 028 patients (74.0%); missing data analysis confirmed a Missing at Random (MAR) pattern, justifying multiple imputation by chained equations (MICE; 20 imputed datasets, Rubin’s Rules, Python v3.8). Model fit was additionally assessed using -2 Log Likelihood (-2LL), Cox & Snell R², and Nagelkerke R² (SPSS v21).

The same discrimination, calibration, and model fit methods were applied to the validation cohort to assess temporal stability of the identified markers.

### Nomogram

A clinical scoring nomogram was constructed using Standard LR coefficients from the complete dataset (n=1, 028) using Python. Each marker was assigned points proportional to its regression coefficient, with total scores mapped to four risk categories. Hypothyroidism was retained in the regression model but excluded from the nomogram as it did not reach significance (OR = 0.715, p=0.092).

## Results

### Baseline characteristics

Of the 1, 028 participants (514 matched pairs), 33% were male. Median age and BMI were 46 (IQR 12) years and 29.7 (IQR 5.5) kg/m², respectively. Obesity Class I (BMI 25.0–29.9 kg/m²) was present in 52% of participants and Obesity Class II (BMI ≥30.0 kg/m²) in 48%. Among diabetic patients, median diabetes duration was 8.5 (IQR 6.6) years.

### Comparison of diabetes vs no diabetes group

The diabetes group showed significantly higher prevalence of insulin resistance (23% vs 12%), systemic inflammation (hsCRP ≥3 mg/L: 62% vs 50%), antihypertensive medication use (33% vs 25%), and dyslipidemia medication use (48% vs 20%) compared to the no-diabetes group (McNemar test, all p<0.001) (**Table 1**). Poor beta-cell function was significantly more prevalent in the diabetes group, while good beta-cell function was more common in the no-diabetes group (p<0.001). Low HDL-C was significantly more prevalent in the diabetes group (72.8% vs 55.1%; OR = 2.2, 95% CI: 1.7–2.8, p=0.001). Hypothyroidism was significantly higher in the no-diabetes group (p=0.001). Regarding BMI category, Obesity Class I was more prevalent in the diabetes group while Obesity Class II was more prevalent in the no-diabetes group (p<0.001).

**Table 1:** Baseline characteristics of matched case-control pairs: diabetes versus no-diabetes groups (n=514 pairs).

Parameter	Diabetes (n=514)	No diabetes (n=514)	Odds ratio	95% CI	p value
Age	46 (12)	46 (12)	–	–	1.000
Sex			1	0.77 – 1.29	0.532
Male	167 (33%)	167 (33%)			
Female	347 (67%)	347 (67%)			
BMI (kg/m^2^)			1.7	1.36 – 2.23	0.392
Obesity Class I (25 to 30)	302 (59%)	231 (45%)	–	–	
Obesity Class II (>30)	212 (41%)	283 (55%)			
Median IQR	29 (5.2)	30.5 (5.4)			<0.001
hsCRP (mg/l)			1.6	1.24 – 2.04	0.005
Inflammation (≥3)	317 (62%)	258 (50%)	–	–	
No inflammation (<3)	197 (38%)	256 (50%)			
Median IQR	4 (6.7)	3.0 (4.4)			<0.001
HOMA2%B			32.4	19.86 - 52.93	<0.001
Poor beta cell function (≤50)	285 (55%)	19 (4%)	–	–	
Good beta cell function (>50)	229 (45%)	495 (96%)			
Median IQR	46.8 (38.4)	104.8 (49.8)			<0.001
HOMA2IR			2.2	1.56 – 3.06	<0.001
Insulin resistance (≥2)	117 (23%)	61 (12%)	–	–	
No insulin resistance (<2)	397 (77%)	453 (88%)			
Median IQR	1.3 (1)	1.1 (0.8)			0.002
On anti-hypertensive medicine			1.5	1.15 – 1.99	<0.001
Yes	171 (33%)	127 (25%)			
No	343 (66%)	387 (75%)			
On dyslipidemia management medication			3.7	2.77 – 4.83	<0.001
Yes	246 (48%)	103 (20%)			
No	268 (52%)	411 (80%)			
Hypothyroidism			0.7	0.51 – 0.93	<0.001
Yes	98 (19%)	130 (25%)			
No	416 (81%)	384 (75%)			
HbA1C (%)	7.7 (2.5)	5.4 (0.30)	–	–	<0.001
Fasting blood glucose (mg/dl)	134 (59.9)	90 (10.2)	–	–	<0.001
Total Cholesterol (mg/dl)	174.9 (55.9)	183.7 (45.2)	–	–	<0.001
HDL-C (mg/dl)	40.1 (12.1)	44 (11.5)	–	–	<0.001
LDL (mg/dl)	110 (51.2)	118 (39.3)	–	–	<0.001
Triglyceride (mg/dl)	135 (87.9)	108 (58.5)	–	–	<0.001
Systolic blood pressure (mmHg)	120 (10)	120 (13)	–	–	0.415
Diastolic blood pressure (mmHg)	80 (7)	80 (6)	–	–	0.013

Data presented as frequency (%) with odds ratio (OR) and 95% confidence interval (CI) for categorical variables, and median (interquartile range) for continuous variables. Categorical variables compared using McNemar test; continuous variables compared using Wilcoxon signed-rank test. BMI, body mass index; HbA1c, glycated haemoglobin; HDL-C, high-density lipoprotein cholesterol; LDL-C, low-density lipoprotein cholesterol; hsCRP, high-sensitivity C-reactive protein; HOMA2%B, Homeostatic Model Assessment of beta-cell function; HOMA2IR, Homeostatic Model Assessment of insulin resistance; IQR, interquartile range.

Subgroup analysis within the no-diabetes group revealed that Obesity Class I non-diabetic patients had significantly worse beta-cell function and greater insulin resistance than their Obesity Class II counterparts (HOMA2%B and HOMA2IR, both p<0.001), despite lower absolute BMI. Conversely, systemic inflammation (hsCRP) was higher in Obesity Class II non-diabetic patients (p<0.001).

Significant differences in diastolic blood pressure and lipid profile were also observed between the diabetes and no-diabetes groups (Wilcoxon test, p=0.013).

Baseline characteristics of the development (n=1, 028) and validation (n=966) cohorts are compared in **Supplementary Table 1**. The cohorts differed significantly in age, sex distribution, diabetes duration, and antihypertensive medication use (p ≤ 0.001), while core metabolic markers (HOMA2%B, HOMA2IR, HDL-C, hsCRP, and BMI) were comparable between cohorts (all p>0.05), confirming metabolic homogeneity despite demographic differences.

### Conditional regression on original dataset (n=1028) (model 1)

**Figure 2:**
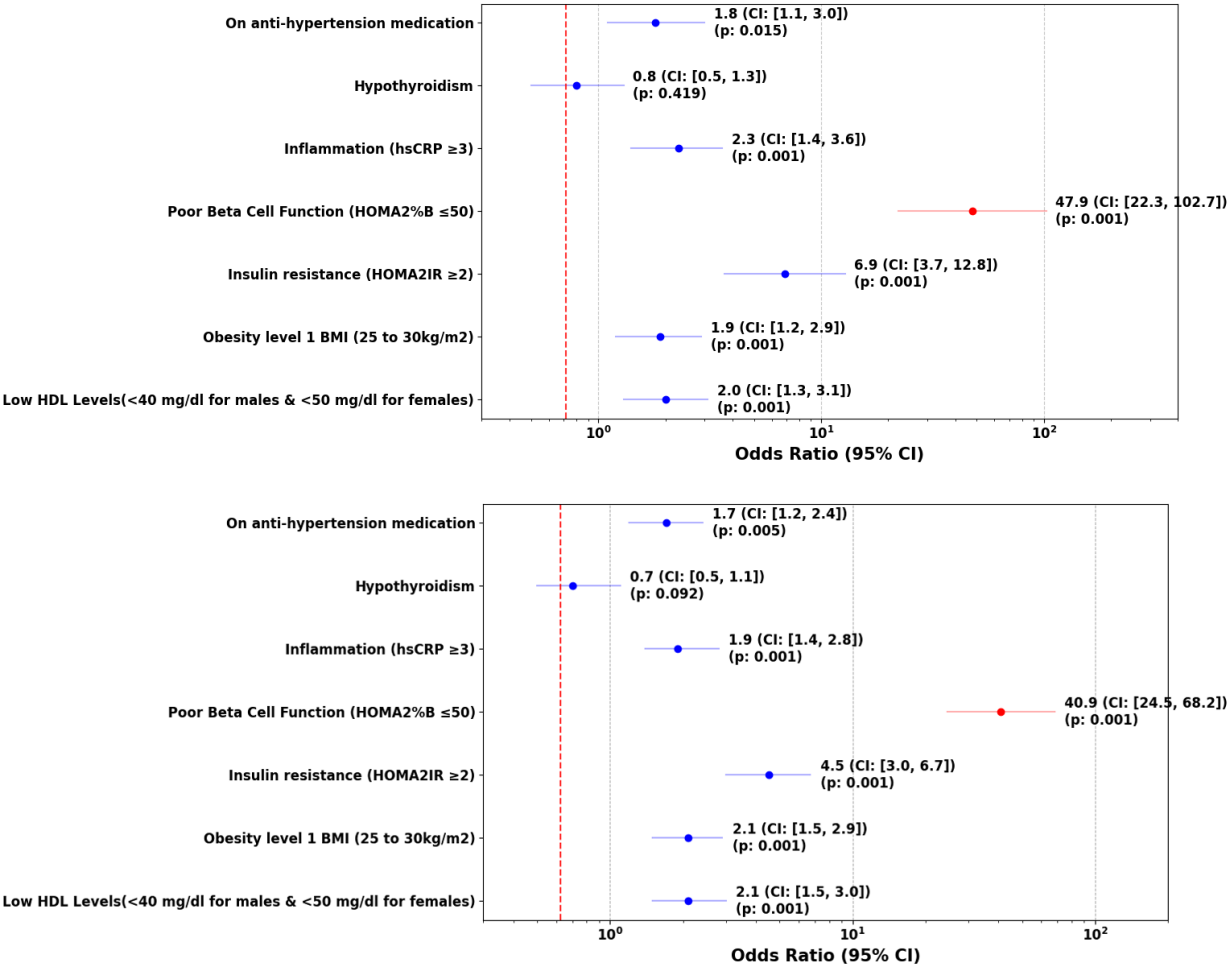
**(A)** Forest plot of adjusted odds ratios from conditional logistic regression (n=1, 028). Blue and red circles represent adjusted odds ratios; horizontal lines represent 95% confidence intervals. Dashed vertical line at 1.0 indicates no effect. P-values are shown in brackets. BMI, body mass index; HDL-C, high-density lipoprotein cholesterol; hsCRP, high-sensitivity C-reactive protein; HOMA2IR, Homeostatic Model Assessment of insulin resistance; HOMA2%B, Homeostatic Model Assessment of beta-cell function. Reference categories: normal HDL-C, Obesity Class II, no insulin resistance, good beta-cell function, no inflammation, no hypothyroidism, no antihypertensive medication. **(B)** Forest plot of adjusted odds ratios from standard binary logistic regression (n=1, 028). Blue and red circles represent adjusted odds ratios; horizontal lines represent 95% confidence intervals. Dashed vertical line at 1.0 indicates no effect. P-values are shown in brackets. BMI, body mass index; HDL-C, high-density lipoprotein cholesterol; hsCRP, high-sensitivity C-reactive protein; HOMA2IR, Homeostatic Model Assessment of insulin resistance; HOMA2%B, Homeostatic Model Assessment of beta-cell function. Reference categories: normal HDL-C, Obesity Class II, no insulin resistance, good beta-cell function, no inflammation, no hypothyroidism, no antihypertensive medication.

Conditional logistic regression (n=1, 028; 514 matched pairs) identified low HDL-C, systemic inflammation, insulin resistance, Obesity Class I, poor beta-cell function, and antihypertensive medication use as significant markers of prevalent T2D. Poor beta-cell function showed the strongest association, with 47.9 times higher odds of T2D compared to good beta-cell function (**Figure 2A**).

### Binary logistic regression on original dataset (n=1028) (model 1)

Standard binary logistic regression was performed to generate predicted probabilities for model performance assessment. The same markers identified in conditional logistic regression were confirmed as significant. Poor beta-cell function again showed the strongest association (OR = 40.9), with all other markers retaining significance in the same direction (**Figure 2B**).

To assess robustness of findings and address potential circularity of HOMA2%B and HOMA2IR, pre-specified sensitivity analyses were performed excluding insulin secretagogue users (Sensitivity A, n=233 excluded), patients with diabetes duration >10 years (Sensitivity B, n=198 excluded), and both groups simultaneously (Sensitivity C). Complete sensitivity analysis results are presented in **Supplementary Table 2**. HOMA2%B and HOMA2IR retained significance across all sensitivity subgroups (OR range 47.4–78.6 and 5.2–6.4 respectively, all p<0.001), confirming robustness of these associations independent of treatment effects or disease duration. Inflammation (hsCRP) demonstrated strengthened associations when secretagogue users and long-standing diabetes were excluded (OR range 2.5–3.7), while HDL-C, antihypertensive medication, and Obesity Class I attenuated to non-significance in sensitivity subgroups, consistent with these associations reflecting advanced disease burden rather than independent biological markers. Additionally, antihypertensive medication retained significance after incorporating systolic blood pressure via MICE imputation (OR = 1.756, 95% CI: 1.045–2.951, p=0.034), confirming the association is not solely explained by underlying blood pressure elevation.

Conditional logistic regression, standard binary logistic regression, and Firth penalized logistic regression were all performed on the complete dataset (n=1, 028) as described in the Statistical Analysis section. All three methods demonstrated consistent direction and significance across all seven markers, confirming robustness of findings regardless of analytical approach (**Supplementary Table 3**).

### Regression on validation data (model 2) (n=966)

**Figure 3:**
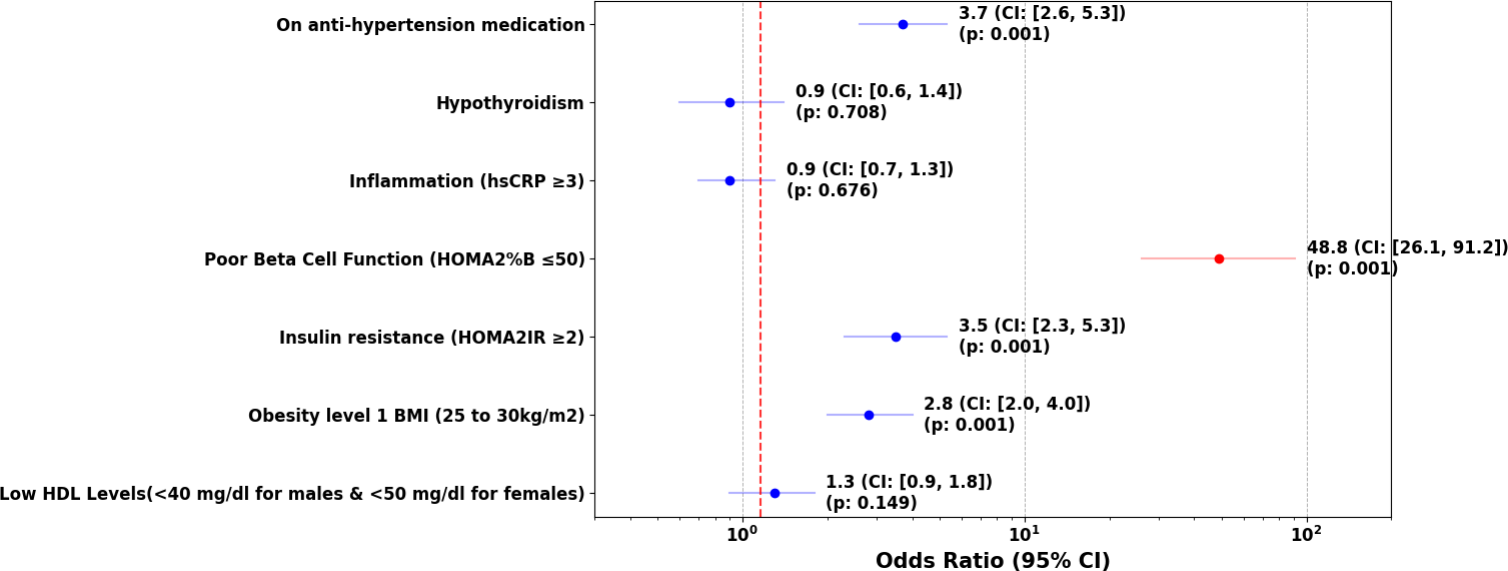
Forest plot of adjusted odds ratios from binary logistic regression on the validation dataset (n=966). Blue and red circles represent adjusted odds ratios; horizontal lines represent 95% confidence intervals. Dashed vertical red line at 1.0 indicates no effect. P-values are shown in brackets. BMI, body mass index; HDL-C, high-density lipoprotein cholesterol; hsCRP, high-sensitivity C-reactive protein; HOMA2IR, Homeostatic Model Assessment of insulin resistance; HOMA2%B, Homeostatic Model Assessment of beta-cell function. Reference categories: normal HDL-C, Obesity Class II, no insulin resistance, good beta-cell function, no inflammation, no hypothyroidism, no antihypertensive medication.

The markers identified in Model 1 were assessed in the independent validation cohort (n=966). Poor beta-cell function retained the strongest association (OR = 48.8), followed by Obesity Class I, antihypertensive medication use, and insulin resistance (**Figure 3**). HDL-C and hsCRP did not reach significance in Model 2, likely reflecting greater within-group heterogeneity in the older, unmatched validation cohort (see Discussion).

### Discrimination, calibration, and decision curve analysis to check performance of models 1 and 2

**Figure 4:**
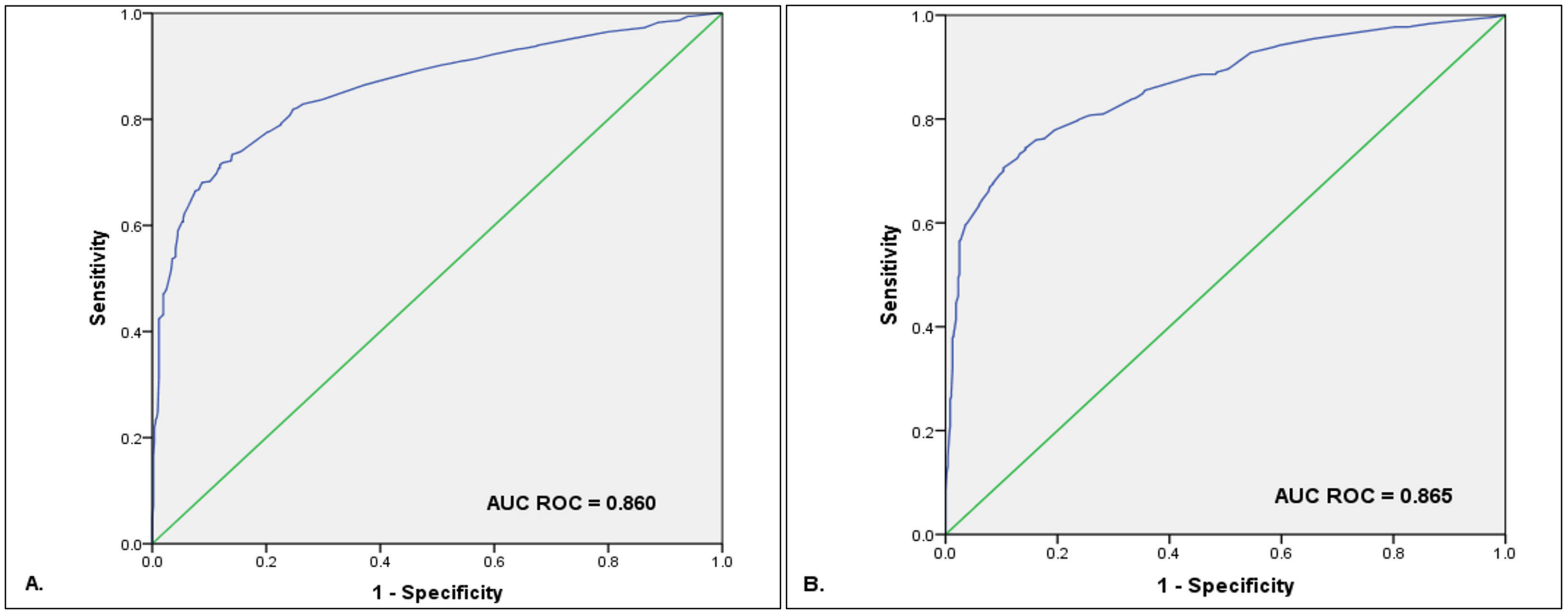
**(A, B)** Receiver operating characteristic (ROC) curves for model 1 (development dataset, n=1, 028) and model 2 (validation dataset, n=966). AUC, area under the curve.

Bootstrap internal validation (1, 000 resamples, Python v3.8) yielded a mean optimism of 0.000 and an optimism-corrected AUC of 0.860, confirming no overfitting (events-per-predictor ratio=73.4). Model 1 achieved AUC ROC of 0.860 (95% CI: 0.837–0.883, p<0.001), indicating good discrimination (**Figure 4A**). At the optimal cut-off of 0.28 (Youden index=0.49), sensitivity was 86.6% (95% CI: 83.4–89.3%) and specificity 62.5% (95% CI: 58.5–66.5%). Model 2 achieved AUC ROC of 0.865 (95% CI: 0.842–0.888, p<0.001), with sensitivity 85.5% (95% CI: 82.1–88.3%) and specificity 64.4% (95% CI: 60.1–68.6%) at cut-off 0.30 (Youden index=0.49) (**Figure 4B**).

**Figure 5:**
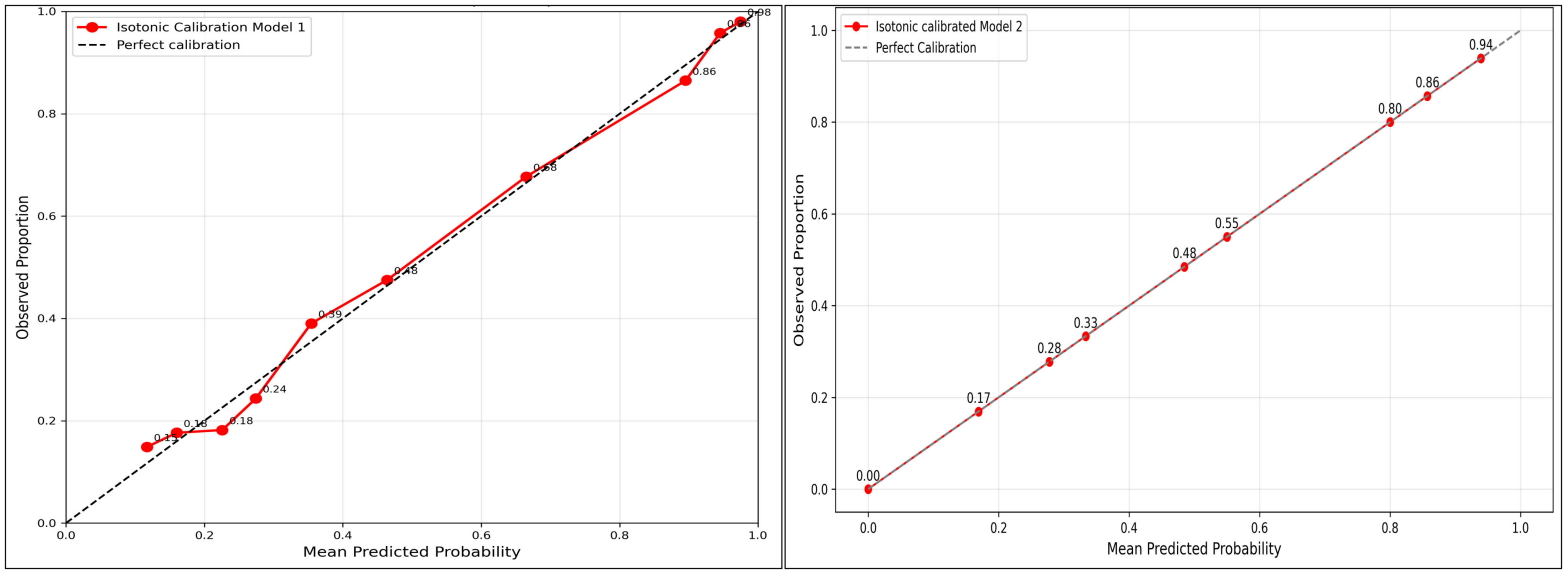
Calibration curve plots for model 1 (development dataset) and model 2 (validation dataset). The curve represents isotonic-calibrated predicted probabilities; the dashed diagonal line represents perfect calibration (ideal agreement between predicted and observed probabilities).

The Hosmer–Lemeshow test confirmed good calibration for both models (Model 1: p=0.562; Model 2: p=0.415). Brier scores were 0.14 (Model 1) and 0.162 (Model 2), indicating acceptable calibration. Calibration plots showed good agreement between predicted and observed probabilities across most risk strata, with minor instability in the low-probability range (0.10–0.20), likely attributable to the bimodal distribution of predicted probabilities inherent to the matched-pair design after isotonic adjustment (**Figure 5**).

Model fit assessed by -2LL was higher in Model 2 (927.045) than Model 1 (615.454), reflecting the larger unmatched validation sample. Model 1 explained 38.4% (Cox & Snell R²) and 51.2% (Nagelkerke R²) of variance; Model 2 explained 39.6% and 52.7% respectively, confirming retained explanatory strength in the validation cohort.

**Figure 6:**
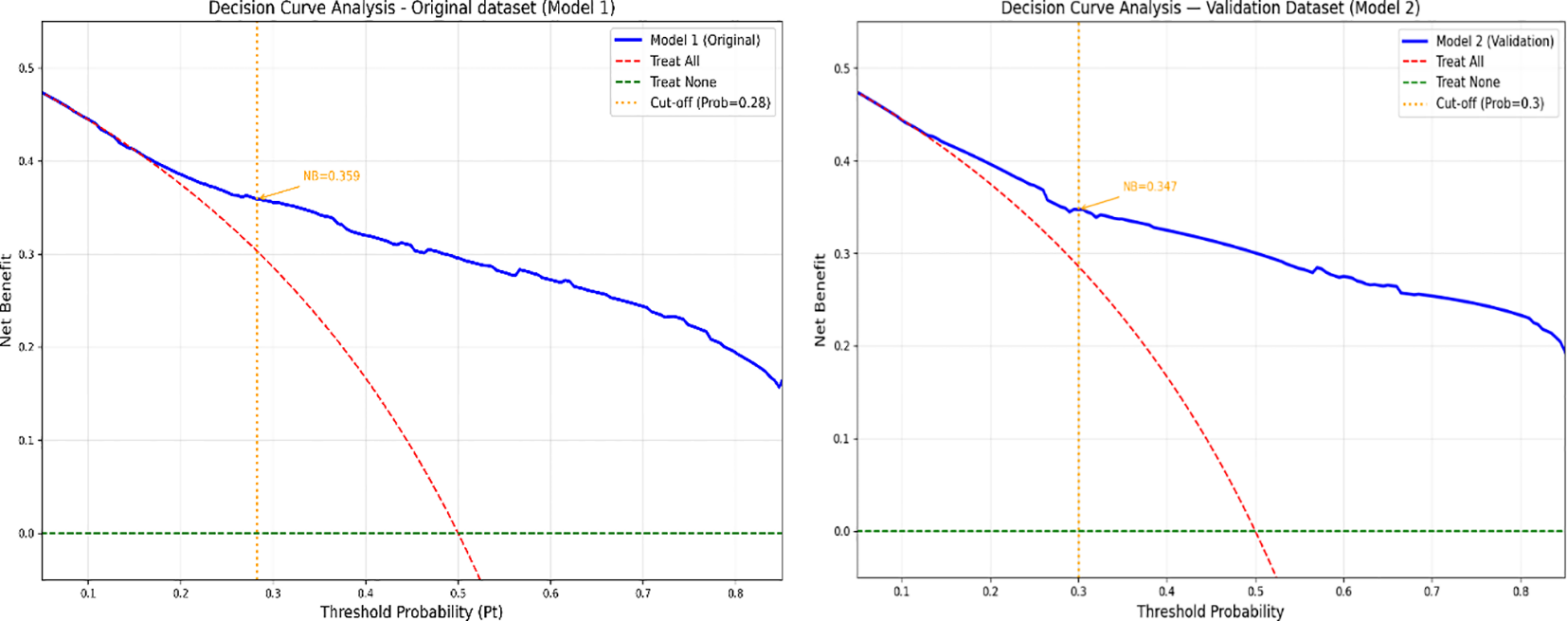
Decision curve analysis for model 1 (development dataset) and model 2 (validation dataset). Net benefit is plotted against threshold probability. The blue solid line represents the logistic regression model; the red dashed line represents the ‘treat-all’ strategy; the green dashed line represents the ‘treat-none’ strategy. The model demonstrates net clinical benefit over both reference strategies across a wide range of threshold probabilities.

Decision curve analysis demonstrated consistent clinical utility across both development and validation cohorts. In Model 1, net benefit of 0.359 was observed at the clinical cut-off of 0.28, outperforming both ‘treat-all’ and ‘treat-none’ strategies across threshold probabilities 0.10–0.85. Model 2 similarly demonstrated net benefit of 0.347 at a threshold of 0.30 (**Figure 6**).

### Nomogram

**Figure 7:**
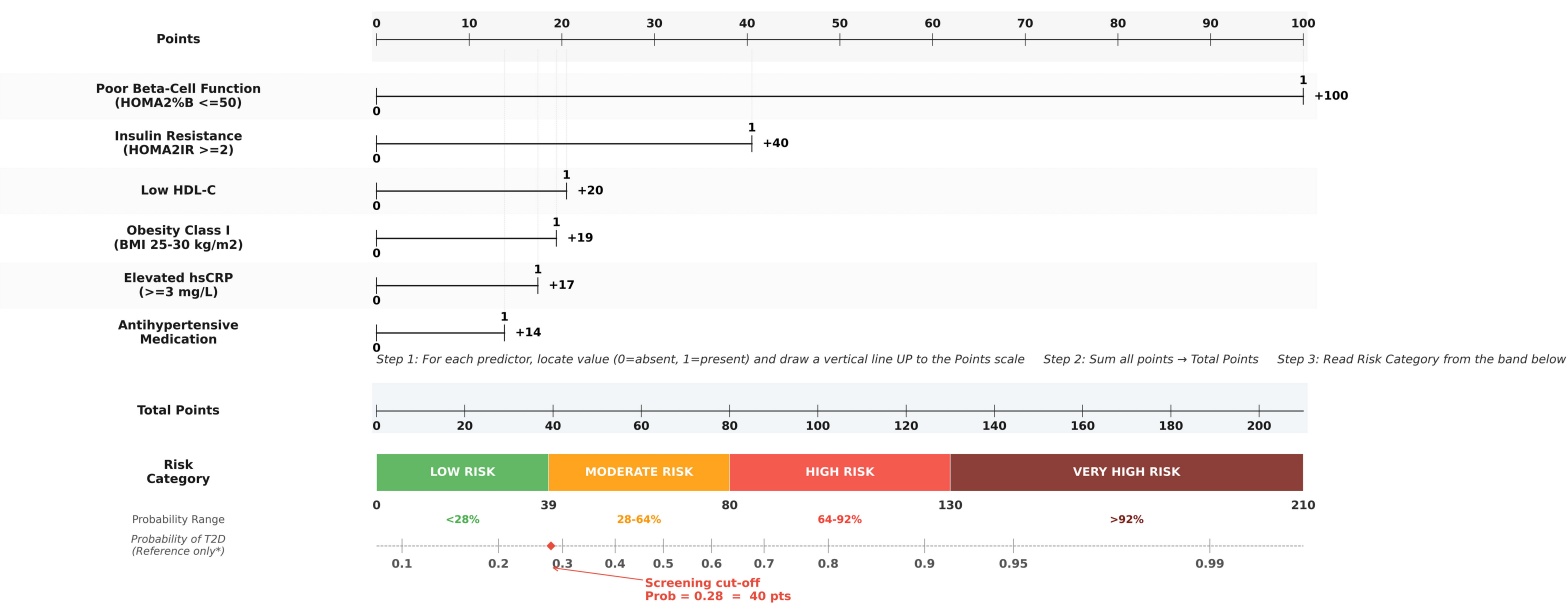
Clinical scoring nomogram for risk stratification of prevalent T2D in obese individuals. Each marker is assigned points proportional to its Standard LR regression coefficient. Total scores (0–212 points) are mapped to four risk categories: Low risk (<40 points, <28% probability), Moderate risk (40–79 points, 28–64%), High risk (80–129 points, 64–92%), and Very High risk (≥130 points, >92%). HDL-C, high-density lipoprotein cholesterol; hsCRP, high-sensitivity C-reactive protein; BMI, body mass index; HOMA2IR, Homeostatic Model Assessment of insulin resistance; HOMA2%B, Homeostatic Model Assessment of beta-cell function. Probability scale spacing is non-linear (logistic function). Use risk category bands above for clinical decision making.

Using Standard LR coefficients from the complete dataset (n=1, 028), each marker was assigned points proportional to its regression coefficient (**Figure 7**): poor beta-cell function (HOMA2%B ≤50) = 100.0 points; insulin resistance (HOMA2IR ≥2) = 40.5 points; low HDL-C = 20.5 points; Obesity Class I (BMI 25.0–29.9 kg/m²) = 19.4 points; elevated hsCRP (≥3 mg/L) = 17.4 points; antihypertensive medication = 13.8 points. Hypothyroidism was retained in the regression model but excluded from the clinical scoring nomogram as it did not reach significance (OR = 0.715, p=0.092). Total scores range from 0 to 212 points, mapped to four risk categories: Low risk (<40 points, <28% probability), Moderate risk (40–79 points, 28–64%), High risk (80–129 points, 64–92%), and Very High risk (≥130 points, >92%).

As a potential future application, contingent on external validation and prospective evaluation, the following risk-stratified actions could be considered based on nomogram total points and corresponding predicted probability of prevalent T2D:

Low risk (<40 points, <28% probability): routine lifestyle counselling, dietary optimisation, and annual metabolic monitoring including fasting glucose and HbA1c.

Moderate risk (40–79 points, 28–64% probability): enhanced structured lifestyle intervention, six-monthly HbA1c and fasting glucose monitoring, targeted dietary modification, and supervised physical activity programme.

High risk (80–129 points, 64–92% probability): comprehensive metabolic assessment including oral glucose tolerance test where indicated, clinical review by an endocrinologist or diabetologist, three-monthly monitoring, and consideration of pharmacological diabetes prevention strategies in accordance with national and international guidelines (25, 26).

Very High risk (≥130 points, >92% probability): urgent clinical evaluation to confirm or exclude a Type 2 diabetes diagnosis, comprehensive workup including HbA1c, fasting and postprandial glucose, and fasting insulin, and immediate initiation of appropriate management if diagnosis is confirmed. These are presented as potential future applications rather than recommendations directly supported by the current study. Given the cross-sectional, retrospective, single-center design, external validation across independent populations and prospective evaluation are required before this tool can be considered for routine clinical practice or before these management actions can be recommended with confidence.

To illustrate clinical application, consider two patients both with Obesity Class I (BMI 25–29.9 kg/m²: +19.4 pts), antihypertensive medication use (+13.8 pts), and low HDL-C (+20.5 pts), with no hsCRP elevation (0 pts). Patient A, with poor beta-cell function (+100.0 pts) and insulin resistance (+40.5 pts), accumulates 194.2 points, corresponding to a predicted probability of 99.2% (Very High Risk, ≥130 points), warranting immediate clinical evaluation. Patient B, without HOMA2 abnormalities (0 pts), accumulates 53.7 points, corresponding to a predicted probability of 39.7% (Moderate Risk, 40–79 points), indicating closer metabolic monitoring. This demonstrates that the tool appropriately stratifies risk across the clinical spectrum and does not default to high risk for all patients.

## Discussion

This study identified and temporally validated key metabolic, inflammatory, and pharmacological markers associated with prevalent T2D in obese individuals using an age-sex matched case-control design. Significant markers included poor beta-cell function, insulin resistance, low HDL-C, elevated hsCRP, Obesity Class I, and antihypertensive medication use, demonstrating consistent direction and significance across both development and validation cohorts. Poor beta-cell function emerged as the strongest discriminative marker, with adjusted odds ratios (AOR) of 40.9 (Standard LR) and 47.9 (Conditional LR) in the development cohort, and 48.8 in the validation cohort. These findings highlight the importance of early beta-cell assessment in obese Indian individuals, a population underrepresented in existing diabetes risk stratification literature.

Of these markers, HOMA2%B and HOMA2IR warrant particular discussion given their derivation from fasting glucose and insulin. The strong associations between HOMA2IR, HOMA2%B, and prevalent T2D are consistent with literature identifying these as central metabolic markers in obesity-related T2D (1, 2, 5). We acknowledge that the cross-sectional design precludes determination of whether these indices antecede or result from T2D, as elevated fasting glucose – a diagnostic criterion – directly decreases HOMA2%B and increases HOMA2IR scores by mathematical construction. The persistence of these associations across all three sensitivity analyses, including after excluding insulin secretagogue users and patients with long-standing disease, supports a genuine biological relationship beyond diagnostic circularity. Beyond HOMA2 indices, the higher prevalence of antihypertensive and dyslipidemia medication use in the diabetes group reflects the well-established clustering of cardiometabolic risk in this population (7, 8, 27, 28), while elevated hsCRP supports the role of chronic low-grade inflammation in obesity-related T2D (6, 7).

While the above metabolic markers were expected findings, the BMI distribution across groups presented a paradox warranting careful interpretation: Obesity Class I was more prevalent among diabetic patients, while Obesity Class II was more prevalent among non-diabetic patients. Subgroup analysis within the no-diabetes group revealed that Obesity Class I non-diabetic patients had significantly worse beta-cell function and greater insulin resistance than their Obesity Class II counterparts (HOMA2%B and HOMA2IR, both p<0.001) despite lower absolute BMI, while systemic inflammation was paradoxically higher in Obesity Class II non-diabetic patients (p<0.05). This dissociation between adiposity and metabolic dysfunction is consistent with the Metabolically Healthy Obese (MHO) phenotype (29), reported in 20–30% of obese individuals in published literature. Three complementary mechanisms may explain this pattern. First, South Asian individuals accumulate visceral adipose tissue and develop beta-cell vulnerability at lower BMI thresholds, predisposing to T2D at Obesity Class I levels (9). Second, survival bias may contribute metabolically susceptible individuals likely develop T2D while still within the Obesity Class I range, before progressing to level 2, selectively enriching the higher BMI pool with metabolically resilient individuals (30). Third, reverse causality cannot be excluded, as post-diagnosis weight loss through osmotic diuresis and dietary modification may have shifted some diabetic patients from level 2 to level 1 BMI at recruitment (31).

A further unexpected observation was the higher prevalence of hypothyroidism in the no-diabetes group. This reflects the known association between hypothyroidism and weight gain, with one plausible mechanism being that hypothyroidism-induced adipose expansion preferentially promotes subcutaneous rather than visceral fat deposition, favoring a metabolically protective distribution associated with preserved insulin sensitivity (32, 33). However, this remains speculative in the context of our cross-sectional design, and the non-significant multivariate result (OR = 0.715, 95% CI: 0.485–1.056, p=0.092) indicates that hypothyroidism does not independently discriminate diabetes status after adjustment for other metabolic markers. This observation is presented as exploratory; future prospective studies incorporating thyroid function and body composition assessment would be required to test this hypothesis formally.

Coming to model performance across cohorts, all markers were replicated in the validation cohort with the exception of HDL-C and hsCRP. As confirmed by the baseline comparison (**Supplementary Table 1**), biomarker distributions were comparable between cohorts (HDL-C p=0.321, hsCRP p=0.988), indicating that attenuation reflects cohort compositional differences rather than systematic biomarker variation. The validation cohort was significantly older, had longer diabetes duration, and included a greater proportion of patients on antihypertensive medication, introducing greater within-group heterogeneity in the unmatched sample that reduces the discriminatory power of moderate-effect-size markers. This is consistent with published evidence that inflammatory and lipid markers show greater variability in treatment-exposed populations (34, 35). HOMA2%B and HOMA2IR retained significance in both cohorts, confirming their central discriminative role.

The slightly higher AOR for poor beta-cell function in the validation cohort (48.8) compared to the development cohort (40.9, Standard LR) is consistent with the older age and longer diabetes duration of the validation sample, which amplifies metabolic separation between groups. Importantly, bootstrap validation yielded an optimism of 0.000, confirming the absence of overfitting and supporting the stability of observed associations. The directionality and significance of beta-cell dysfunction across both datasets confirm its central discriminative role; future studies should assess its associations through prospective longitudinal designs to establish temporal directionality.

Comprehensive model evaluation confirmed good performance across all metrics. AUC ROC values indicated good discrimination in both cohorts (0.860 development, 0.865 validation). Calibration was supported by non-significant Hosmer–Lemeshow tests (p=0.562 Model 1, p=0.415 Model 2), Brier scores of 0.14 and 0.162 respectively, and calibration plots. Cox & Snell R² and Nagelkerke R² confirmed substantial explained variance. Decision curve analysis demonstrated net clinical benefit across clinically relevant threshold probabilities in both cohorts, supporting practical utility beyond statistical performance.

To translate these findings into a clinically actionable format, a scoring nomogram was developed assigning weighted points to each marker based on Standard LR coefficients. Clinicians can rapidly estimate an individual’s probability of prevalent T2D by summing scores across accessible clinical and biochemical parameters. Compared to existing Indian diabetes risk instruments such as the Indian Diabetes Risk Score (IDRS) (36) and the Ramachandran score (37), the present tool provides a more comprehensive assessment by incorporating beta-cell function, insulin resistance, inflammatory, and pharmacological markers alongside anthropometric parameters. The tool demonstrated good discrimination (AUC 0.860) with 86.6% sensitivity (95% CI: 83.4–89.3%) and 62.5% specificity (95% CI: 58.5–66.5%). However, as the model identifies markers associated with prevalent T2D in a retrospective, cross-sectional, single-center cohort of obese individuals enrolled in a lifestyle intervention program, it cannot be considered a risk prediction tool for incident diabetes. External validation across independent populations and prospective evaluation are required before this nomogram can be considered for routine clinical application.

The key strengths of this study include a rigorously matched case-control design, temporal internal validation on an independent time-cohort, and comprehensive model evaluation encompassing discrimination, calibration, and decision curve analysis. The multifactorial approach incorporating metabolic, inflammatory, and pharmacological markers advances diabetes risk stratification beyond traditional single-risk factor models, addressing a critical gap in the Indian obesity literature.

This study has several limitations. First, the cross-sectional retrospective design means that the direction of associations cannot be established; whether identified markers preceded or resulted from T2D remains unclear. In particular, HOMA2%B and HOMA2IR are directly influenced by the hyperglycemia of established diabetes and should therefore be interpreted as markers of the diabetic metabolic state rather than independent predictors of future onset. Prospective longitudinal studies are needed to clarify these temporal relationships. Second, although cases and controls were matched for age and sex, other important confounders such as diet, physical activity, and genetic factors were not measured and may have influenced the results. Also, antihypertensive medication use should be interpreted as a marker of overall cardiovascular disease burden rather than a direct biological cause of diabetes. Third, all participants were recruited from a single private lifestyle clinic in urban India, which may not be representative of the broader Indian population, particularly those in rural areas or lower-income groups. The validation cohort came from the same center at a later time period; independent validation at other centers across India is needed before these findings can be applied more broadly. Fourth, the nomogram requires a fasting insulin test to calculate HOMA2%B and HOMA2IR, which may not be available in resource-limited settings. When these two markers were removed, the simplified model performed poorly (AUC = 0.676), confirming that fasting insulin measurement is essential for reliable risk assessment (**Supplementary Table 4**). Future studies should explore alternative approaches such as HbA1c-based or C-peptide-based indices that do not require fasting insulin. Fifth, of 13, 066 patients screened, 6, 459 (49.4%) were excluded due to missing first-consultation values for fasting insulin and hsCRP. As all enrolling patients undergo the same standardized investigation panel irrespective of diabetes status, missingness reflects visit compliance rather than differential clinical decision-making, consistent with a Missing Completely at Random (MCAR) pattern. Blood pressure data were additionally unavailable for 267 of 1, 028 included patients, showing a Missing at Random (MAR) pattern; multiple imputation by chained equations (MICE) was applied to address this. Finally, future research incorporating genetic data, dietary patterns, and diverse populations across different regions and ethnicities will strengthen the generalizability and clinical utility of these findings.

In conclusion, this study identified and temporally validated key metabolic, inflammatory, and pharmacological markers associated with prevalent T2D in obese Indians, with poor beta-cell function emerging as the strongest discriminative marker. A clinical scoring nomogram was developed as a discrimination tool for prevalent T2D in this population, requiring external validation and prospective evaluation before consideration for routine clinical practice. These findings contribute to a growing evidence base for targeted diabetes prevention in high-risk obese populations, and future studies incorporating longitudinal designs, genetic data, and diverse geographic populations will further strengthen the generalizability and clinical utility of these findings.

## Data Availability

The raw data supporting the conclusions of this article will be made available by the authors, without undue reservation.
