# Supplementary material for: Metabolic and clinical markers of T2D in obese Indians: an age-sex matched case-control study
Source: Front Clin Diabetes Healthc. 2026 Aug 5;7:1809646. doi: 10.3389/fcdhc.2026.1809646 (PMC13485558; doi:10.3389/fcdhc.2026.1809646)
Supplement: Supplementary file 1 [file DataSheet1.docx]

| **Parameter** | **Original**  **(n=1028)** | **Validation**  **(n=966)** | ***p* value** |
| --- | --- | --- | --- |
|  |  |  |  |
| Age | 46 (12) | 47 (14) | <0.001 |
| Sex  Male  Female | 334 (32%)  694 (68%) | 426 (44%)  540 (56%) | <0.001 |
| BMI (kg/m^2^)  25 to 30  >30  Median IQR | 533 (52%)  495 (48%)  29.8 (5.5) | 504 (52%)  462 (48%)  29.8 (5.3) | 0.884 |
| hsCRP (mg/l)  Inflammation (≥3)  No inflammation (<3)  Median IQR | 575 (56%)  453 (44%)  3.5 (5.3) | 540 (56%)  426 (44%)  3.4 (4.7) | 0.988 |
| HOMA2%B  Poor beta cell function (≤50)  Good beta cell function (>50)  Median IQR | 304 (30%)  724 (70%)  78.4 (71) | 266 (28%)  700 (72%)  80 (68) | 0.315 |
| HOMA2IR  Insulin resistance (≥2)  No insulin resistance (<2)  Median IQR | 178 (17%)  850 (83%)  1.2 (1.0) | 161 (17%)  805 (83%)  1.2 (0.9) | 0.700 |
| On anti-hypertensive medicine  Yes  No | 298 (29%)  730 (71%) | 346 (36%)  620 (64%) | 0.001 |
| On dyslipidemia management medication  Yes  No | 349 (34%)  679 (66%) | 346 (36%)  620 (64%) | 0.382 |
| Hypothyroidism  Yes  No | 228 (22%)  800 (78%) | 202 (21%)  764 (79%) | 0.491 |
| Diabetes  Yes  No | 514 (50%)  514 (50%) | 483 (50%)  483 (50%) | 1.000 |
| Duration | 8.5 (6.6) | 10.3 (10.3) | <0.001 |
| HbA1C (%) | 5.6 (2.3) | 5.7 (2.3) | 0.458 |
| Fasting blood glucose (mg/dl) | 97.1 (45) | 98 (44) | 0.962 |
| HDL-C (mg/dl) | 43 (12.1) | 42 (11) | 0.321 |
| Systolic blood pressure (mmHg) | 120 (12) | 120 (10) | 0.012 |
| Diastolic blood pressure (mmHg) | 80 (6) | 80 (4) | 0.263 |

**Supplementary Table 1. Baseline characteristics of the development (n=1,028) and validation (n=966) cohorts**

*Data presented as frequency (%) for categorical variables and median (interquartile range) for continuous variables. p-values calculated using chi-square test for categorical variables and Mann-Whitney U test for continuous variables. BMI, body mass index; HbA1c, glycated haemoglobin; HDL-C, high-density lipoprotein cholesterol; hsCRP, high-sensitivity C-reactive protein; HOMA2%B, Homeostatic Model Assessment of beta-cell function; HOMA2IR, Homeostatic Model Assessment of insulin resistance.*

**Supplementary Table 2: Pre-specified sensitivity analyses — conditional logistic regression across subgroups (n=1,028)**

| Predictor | Full Dataset  (n=1,028) | | Sens A: Excl.  Secretagogues | | Sens B: Excl.  DM >10 years | | Sens C: Excl.  Both | |
| --- | --- | --- | --- | --- | --- | --- | --- | --- |
|  | **OR (95% CI)** | **p-value** | **OR (95% CI)** | **p-value** | **OR (95% CI)** | **p-value** | **OR (95% CI)** | **p-value** |
| Poor beta-cell function (HOMA2%B ≤50) | 47.9 (22.3–102.7) | **<0.001** | 50.9 (16.7–154.9) | **<0.001** | 78.6 (22.4–276.2) | **<0.001** | 47.4 (12.8–175.7) | **<0.001** |
| Insulin resistance (HOMA2IR ≥2) | 6.9 (3.7–12.8) | **<0.001** | 6.0 (2.9–12.5) | **<0.001** | 6.5 (3.1–13.3) | **<0.001** | 5.2 (2.4–11.4) | **<0.001** |
| Low HDL-C | 1.9 (1.3–3.1) | **0.003** | 1.4 (0.8–2.5) | 0.222 | 1.7 (0.9–3.0) | 0.066 | 1.5 (0.8–2.9) | 0.235 |
| Inflammation (hsCRP ≥3 mg/L) | 2.3 (1.4–3.6) | **<0.001** | 2.5 (1.4–4.7) | **0.004** | 3.7 (1.9–7.0) | **<0.001** | 3.6 (1.6–7.7) | **0.001** |
| Antihypertensive medication | 1.8 (1.1–3.0) | **0.015** | 1.3 (0.7–2.5) | 0.433 | 1.2 (0.6–2.2) | 0.589 | 1.3 (0.6–2.8) | 0.53 |
| Hypothyroidism | 0.8 (0.5–1.3) | 0.419 | 0.8 (0.4–1.5) | 0.506 | 0.6 (0.3–1.2) | 0.137 | 0.5 (0.2–1.2) | 0.128 |
| Obesity Class I | 1.8 (1.2–2.9) | **0.007** | 1.6 (0.9–2.8) | 0.097 | 1.9 (1.1–3.3) | **0.028** | 1.5 (0.8–2.9) | 0.195 |

*All analyses use conditional logistic regression on the complete dataset (n=1,028; 514 matched pairs), implemented in Python v3.8. Sens A excludes patients on insulin secretagogue medications (sulfonylureas and meglitinides; n=233 removed). Sens B excludes patients with diabetes duration >10 years (n=198 removed). Sens C excludes both groups simultaneously. OR, odds ratio; CI, confidence interval. Bold p-values indicate statistical significance (p<0.05)*

**Supplementary Table 3:** **Comparison of standard, conditional, and Firth penalised logistic regression — complete dataset (n=1,028)**

| Predictor | Standard LR | | Conditional LR | | Firth penalized LR | |
| --- | --- | --- | --- | --- | --- | --- |
|  | **OR (95% CI)** | **p-value** | **OR (95% CI)** | **p-value** | **OR (95% CI)** | **p-value** |
| Poor beta-cell function (HOMA2%B ≤50) | 40.9 (24.5–68.2) | **<0.001** | 47.9 (22.3–102.7) | **<0.001** | 38.9 (23.4–64.6) | **<0.001** |
| Insulin resistance (HOMA2IR ≥2) | 4.5 (3.0–6.7) | **<0.001** | 6.9 (3.7–12.8) | **<0.001** | 4.4 (2.9–6.6) | **<0.001** |
| Low HDL-C | 2.1 (1.5–3.0) | **<0.001** | 1.9 (1.3–3.1) | **0.003** | 2.1 (1.5–2.9) | **<0.001** |
| Inflammation (hsCRP ≥3 mg/L) | 1.9 (1.4–2.7) | **<0.001** | 2.3 (1.4–3.6) | **<0.001** | 1.9 (1.4–2.7) | **<0.001** |
| Antihypertensive medication | 1.7 (1.2–2.4) | **0.005** | 1.8 (1.1–3.0) | **0.015** | 1.7 (1.2–2.4) | **0.005** |
| Hypothyroidism | 0.7 (0.5–1.1) | 0.092 | 0.8 (0.5–1.3) | 0.419 | 0.7 (0.5–1.1) | 0.093 |
| BMI (25 to 30kg/m^2^) | 2.1 (1.5–2.9) | **<0.001** | 1.9 (1.2–2.9) | **0.007** | 2.0 (1.4–2.9) | **<0.001** |

*Standard LR: standard binary logistic regression; primary model for predicted probability generation (n=1,028). Conditional LR: conditional logistic regression accounting for the matched-pair structure (514 pairs, Python v3.8); cannot generate absolute predicted probabilities due to absence of intercept term. Firth LR: Firth penalised logistic regression implemented using the Jeffreys invariant prior penalty (scipy.optimize, Python v3.8); confirmatory sensitivity analysis for quasi-complete separation in HOMA2%B. All models use the complete dataset (n=1,028). OR, odds ratio; CI, confidence interval. Bold p-values indicate statistical significance (p<0.05)*

**Supplementary Table 4: Logistic Regression removing HOMA2 estimates**

| Predictor | Standard LR | | Coefficient | Nomogram points allocated |
| --- | --- | --- | --- | --- |
|  | **OR (95% CI)** | **p-value** |  |  |
| Low HDL-C | 2.28 (1.73–2.98) | **<0.001** | **0.822** | **100** |
| Inflammation (hsCRP ≥3 mg/L) | 1.91 (1.45–2.50) | **<0.001** | **0.646** | **78.5** |
| Antihypertensive medication | 1.73 (1.30–2.31) | **<0.001** | **0.550** | **66.9** |
| Hypothyroidism | 0.66 (0.48–0.90) | 0.009 | -0.414 | 0 |
| BMI (25 to 30kg/m^2^) | 2.20 (1.68–2.88) | **<0.001** | **0.789** | **96** |

*OR = Odds Ratio; CI = Confidence Interval. Nomogram points derived by scaling each coefficient relative to the maximum positive coefficient (Low HDL-C = 100 points; point scale factor = 121.65). Thyroid hormone replacement is protective (OR<1); absence of thyroid medication contributes 50.4 points while presence contributes 0 points. This simplified five-predictor model (excluding HOMA2%B and HOMA2IR) achieved* ***AUC=0.676****, below the acceptable discrimination threshold, confirming the clinical necessity of fasting insulin measurement for reliable T2D risk stratification*
